# Effects of Dietary 5-Aminolevulinic Acid Supplementation on Laying Performance, Immune Function, Intestinal Microbiota, and Serum Metabolomics in Laying Hens

**DOI:** 10.3390/ani16203173

**Published:** 2026-10-09

**Authors:** Xianci Yao, Chenkai Pan, Kaitian Zheng, Yonghao Shi, Tao Sun, Caimei Yang

**Affiliations:** College of Animal Science and Technology, College of Veterinary Medicine, Zhejiang A&F University, Hangzhou 311300, China; 15639190344@163.com (X.Y.); h3103149957@163.com (C.P.); 19817120570@163.com (K.Z.); 19157736914@163.com (Y.S.); tsun@zafu.edu.cn (T.S.)

**Keywords:** 5-aminolevulinic acid, cecal microbiota, laying hens, metabolism

## Abstract

5-Aminolevulinic acid (5-ALA), a naturally occurring non-proteinogenic amino acid and a key precursor in heme biosynthesis, has attracted increasing attention as a potential functional feed additive in animal production. This study investigated the effects of dietary 5-ALA supplementation on 960 Fengda No. 1 laying hens (42 weeks of age) randomly assigned to four dietary treatments with 16 replicates per treatment (15 birds per replicate) and fed diets supplemented with 0, 250, 500, or 1000 mg/kg 5-ALA for 60 days. Results show that 500 mg/kg 5-ALA improved laying performance and enhanced liver antioxidant capacity (T-AOC, SOD, GSH-Px). It also enhanced serum immunoglobulin concentrations and reduced the concentrations of pro-inflammatory cytokines, including TNF-α, IL-1β, and IL-6. Furthermore, 5-ALA supplementation was associated with changes in the abundances of specific cecal bacterial taxa. Metabolomic analysis revealed that vitamin B6 metabolism showed the highest differential abundance score, with increased 4-pyridoxic acid and 4-pyridoxolactone following 5-ALA supplementation. Collectively, these findings suggest that dietary 5-ALA, particularly at 500 mg/kg, has the potential to improve laying performance and physiological homeostasis, potentially through the modulation of antioxidant and immune functions, gut microbial composition, and metabolic pathways.

## Simple Summary

5-Aminolevulinic acid (5-ALA) is a naturally occurring compound involved in heme biosynthesis and has potential as a functional feed additive for poultry. However, its effects on laying hens remain unclear. In this study, 960 laying hens were fed diets supplemented with 0, 250, 500, or 1000 mg/kg 5-ALA for 60 days. Dietary supplementation with 500 mg/kg 5-ALA improved laying performance and enhanced antioxidant status, as indicated by increased liver antioxidant capacity. It also increased serum immunoglobulin concentrations and reduced pro-inflammatory cytokines, suggesting improved immune function. In addition, metabolomic analysis further revealed that vitamin B6 metabolism was significantly affected, accompanied by increased levels of 4-pyridoxic acid and 4-pyridoxolactone. Overall, dietary 5-ALA may have the potential to improve laying performance and physiological homeostasis by modulating antioxidant and immune responses, gut microbiota, and metabolic pathways in laying hens.

## 1. Introduction

Egg production represents an essential component of the global animal protein supply and plays a critical role in meeting the increasing demand for high-quality nutritional resources [1]. However, the intensification of poultry production systems has imposed considerable challenges on laying hen health and productivity [2]. In particular, laying hens experience substantial physiological stress during peak and late laying periods, during which oxidative imbalance, immune dysfunction, and intestinal disturbances may negatively affect productive performance and egg quality [3].

Oxidative stress can be induced by heat stress [4], high production pressure, dietary factors [5], or disease challenges, leading to reduced laying performance, diminished egg quality, such as decreased eggshell strength and Haugh unit values, and liver damage. The Haugh unit is an established indicator of internal egg quality that primarily reflects albumen quality [5,6]. Alterations in gut microbial communities may compromise intestinal barrier integrity, promote inflammatory responses, and subsequently impair nutrient absorption and metabolic regulation [7]. Therefore, identifying effective nutritional interventions capable of simultaneously improving antioxidant capacity, immune function, and intestinal homeostasis is of considerable interest in poultry production.

5-Aminolevulinic acid (5-ALA), a naturally occurring non-proteinogenic amino acid, acts as a crucial intermediate in porphyrin synthesis [8]. In eukaryotic cells, endogenous 5-ALA is synthesized in mitochondria by 5-aminolevulinate synthase (ALAS) from glycine and succinyl-CoA and serves as the first committed precursor in haem biosynthesis [9]. Through sequential enzymatic reactions, 5-ALA is converted into protoporphyrin IX, which subsequently incorporates iron to generate heme [10]. As an essential component of numerous hemoproteins, heme participates in oxygen transport, electron transfer, mitochondrial energy metabolism, and antioxidant defense systems [11]. Consequently, exogenous 5-ALA supplementation may influence physiological processes associated with oxidative metabolism, immune regulation, and productive performance [12].

Previous studies have demonstrated that dietary 5-ALA supplementation can improve hematological characteristics, iron metabolism, antioxidant capacity, and immune responses in livestock species [13]. In piglets, dairy cattle, and poultry, dietary 5-ALA has been demonstrated to increase growth rate in piglets [14], improve serum iron and hemoglobin levels [15], enhance antioxidant capacity [16], and improve both the egg production and quality of livestock products [17]. However, despite increasing interest in the biological functions of 5-ALA, its effects on laying hens, particularly its influence on intestinal microbial communities and host metabolic profiles, remain poorly understood.

Therefore, the present study was conducted to investigate the effects of dietary 5-ALA supplementation on laying performance, antioxidant status, immune responses, intestinal morphology, cecal microbiota, and metabolomic profiles in laying hens.

## 2. Materials and Methods

### 2.1. Animal Experimentation Methods

All experimental procedures were approved by the Experimental Animal Ethics Committee of Zhejiang A&F University (Approval No. ZAFUAC2025104). For this experiment, 960 healthy laying hens (42 weeks of age) exhibiting comparable egg production were selected from a larger flock and then randomly divided into four groups. Each treatment consisted of 16 replicates, with 15 hens housed in one cage per replicate. Before the dietary treatments commenced, the hens underwent a 3-day adaptation period under experimental housing conditions. During the experimental period, the ambient temperature was maintained at 20 to 22 °C, with a lighting program of 16L/8D. Feed and water were provided ad libitum throughout the experimental period. The feeding regimens were as follows: basal diet without any supplements (CON), basal diet supplemented with 250 mg/kg 5-aminolevulinic acid (A250), 500 mg/kg 5-ALA (A500) and 1000 mg/kg 5-ALA (A1000). The 5-ALA used in this experiment was provided by Zhejiang Vegamax Biotechnology Co., Ltd. (Anji, China). Table 1 presents the composition and nutrient levels of the basal diet formulated according to NRC and Chinese standards (NRC, 1994) [18].

### 2.2. Sample Collection

According to the predetermined laboratory sampling plan, on day 60, 16 hens from each group were randomly selected and weighed. Throughout the 60-day experimental period, egg production rate and egg weight were recorded daily, whereas egg quality was evaluated at weekly intervals. We randomly selected 8 laying hens from each group of 16 for euthanasia by cervical dislocation and sample collection. Serum was obtained from jugular blood samples following centrifugation at 4000× *g* for 10 min at 4 °C. The jejunum and ileum were delineated by Meckel’s diverticulum, and 1 cm segments were harvested from the midpoint of each intestinal segment. The samples were then rinsed with 0.9% NaCl solution and fixed in 4% formaldehyde. The remaining intestinal segments were longitudinally incised, and their contents were rinsed with phosphate-buffered saline (PBS). The mucosal surface was then gently scraped using a sterile glass slide to collect mucosal samples. Cecal contents were collected for microbiological analysis and determination of VFAs, and all harvested samples were stored at −80 °C.

### 2.3. Determination of Laying Performance and Egg Quality

Daily egg production and egg weight were recorded for each replicate according to the method described by Wang et al. [19]. During the experimental period, 8 eggs were randomly collected from each group every week for egg quality determination. Based on the experimental records, the laying rate and average egg weight of laying hens in each experimental group were compiled and statistically analyzed. Egg quality parameters were measured using an egg quality analyzer (Robotmation, Tokyo, Japan) according to the manufacturer’s instructions.

$$\mathrm{Average}\;\mathrm{egg}\;\mathrm{weight}=\frac{\sum_{i=1}^{60}Ai}{60}=\frac{A1+A2+\cdots +A60}{60}\;\left(A_{i}=\mathrm{Average daily egg weight}\right)$$

$$\mathrm{Average}\;\mathrm{egg}\;\mathrm{production}\;\mathrm{rate}=\frac{\sum_{i=1}^{60}Ai}{60}=\frac{A1+A2+\cdots +A60}{60}\;\left(A_{i}=\mathrm{Average daily egg production rate}\right)$$

### 2.4. Biochemical Evaluation

The assessed immunoglobulin indicators included immunoglobulin Y (IgY), immunoglobulin A (IgA), and immunoglobulin M (IgM). Inflammatory markers measured were interleukin-1β (IL-1β), interleukin-6 (IL-6), interleukin-10 (IL-10), and tumor necrosis factor-α (TNF-α). The evaluated antioxidant parameters comprised malondialdehyde (MDA), superoxide dismutase (SOD), glutathione peroxidase (GSH-Px), and total antioxidant capacity (T-AOC). Immune and antioxidant functions were both determined using commercial kits (Nanjing Jiancheng Bioengineering Institute, Nanjing, China) following the manufacturer’s instructions.

### 2.5. Liver Indicators

Approximately 0.2 g of liver tissue was harvested and immediately stored in liquid nitrogen. Liver samples were homogenized in 9 volumes of ice-cold saline on ice with an electric homogenizer until no visible tissue particles remained. The homogenate was centrifuged at 4 °C and 4000× *g* for 10 min, and the resulting supernatant was taken for subsequent assays. The levels of T-AOC, SOD, GSH-Px and MDA content in the liver were measured with commercial kits (Nanjing Jiancheng Bioengineering Institute, Nanjing, China) per the manufacturer’s instructions.

### 2.6. Intestinal Morphological Analysis

According to the method of Chen et al. [20]. The intestinal morphology was evaluated according to the manufacturer’s instructions (Biossci, Changsha, China) using the hematoxylin-eosin staining method. The stained sections were examined under a light microscope, and representative images were captured for subsequent analysis. For each section, eight intact villi and their corresponding crypts were randomly selected for the measurement of villus height (VH) and crypt depth (CD) 3DHISTEC, Budapest, Hungary). The villus-height-to-crypt-depth ratio (VH/CD) was calculated accordingly.

### 2.7. Analysis of Volatile Fatty Acids

Consistent with the previous methodology [21], headspace sampling gas chromatography (Agilent Technologies, Beijing, China) was employed to determine the concentration of volatile fatty acid (VFA) in cecal contents. Briefly, 1 g of cecal content was homogenized with 1 mL of double-distilled water (ddH_2_O). Following centrifugation, 1 mL of supernatant was combined with 0.2 mL of 25% metaphosphoric acid. Analysis of VFA concentrations was performed using an Agilent 1890 Network System (Agilent Technologies, Santa Clara, CA, USA) equipped with a 30 m × 0.25 mm × 0.25 μm capillary column and a flame ionization detector (Agilent Technologies, Santa Clara, CA, USA).

### 2.8. 16S rRNA Sequencing of Microflora in Cecum Contents

According to the method described by Cuccato [22], a random subset of six samples from each treatment group was selected for subsequent 16S rRNA gene sequencing. Total microbial DNA was extracted from cecal contents using the E.Z.N.A. DNA Kit (Omega Bio-tek, Norcross, GA, USA). The V3 to V4 region of the bacterial 16S rRNA gene was amplified using the 338F and 806R primers, and the resulting amplicons were subjected to high-throughput sequencing. Raw sequencing reads were quality filtered using fastp v0.19.6 and merged using FLASH v1.2.11. Amplicon sequence variants (ASVs) were generated using QIIME2 (version 2024.10), and taxonomic classification was performed against the SILVA 16S rRNA reference database using the Naive Bayes classifier. Alpha diversity indices were calculated using Mothur v1.30.2. Beta diversity was assessed based on unweighted UniFrac distances using QIIME v1.9.1, and the resulting distance matrix was visualized by principal coordinates analysis (PCoA). Microbial community composition and relative abundance were further analyzed using R software (version 3.3.1). Differentially abundant taxa among treatment groups were identified using STAMP v2.1.3.

### 2.9. Metabolomics

According to the method of Xu et al. [23], eight samples were randomly selected from each group for the detection of metabolomics. Metabolomic profiling was carried out by Majorbio Bio-Pharm Technology Co., Ltd. (Shanghai, China). Next, 100 μL of serum was mixed with 400 μL of acetonitrile (1:1, *v*/*v*) containing internal standards. After vortexing for 30 s, the samples were subjected to low-temperature ultrasonication at 5 °C for 30 min, followed by incubation at −20 °C for 30 min and centrifugation at 13,000× *g* for 15 min at 4 °C. The supernatants were collected, dried under nitrogen, and reconstituted in 100 μL of acetonitrile (1:1, *v*/*v*). After ultrasonication for 5 min and centrifugation at 13,000× *g* for 10 min at 4 °C, the supernatants were transferred to autosampler vials for analysis.

Untargeted metabolomics analysis was performed by Majorbio Bio-Pharm Technology Co., Ltd. (Shanghai, China) using a UHPLC Orbitrap Exploris 240 system equipped with an HSS T3 column (100 mm × 2.1 mm, 1.8 μm). The injection volume was 3 μL, with a flow rate of 0.40 mL/min and a column temperature of 40 °C. Mobile phase A consisted of water/acetonitrile containing 0.1% formic acid (95:5, *v*/*v*), while mobile phase B consisted of acetonitrile/isopropanol/water containing 0.1% formic acid (47.5:47.5:5, *v*/*v*/*v*). Data were acquired in both positive and negative ion modes over an m/z range of 70 to 1050. Quality control samples were prepared by pooling equal volumes of all samples and were periodically analyzed to monitor the stability and reproducibility of the analytical system.

Raw LC MS data were processed using Progenesis QI (Waters Corporation, Milford, MA, USA) for baseline filtering, peak detection, integration, retention time correction, and peak alignment. Metabolite identification was performed by matching MS and MS/MS data against the Human Metabolome Database, Metlin, and the Majorbio in-house database. Variables detected in at least 80% of samples in at least one group were retained, missing values were replaced by the minimum observed value, and sum normalization was applied. Variables with a relative standard deviation greater than 30% in QC samples were excluded, followed by log10 transformation. Differential metabolites were identified based on VIP > 1 and Student’s *t* test *p* < 0.05. Differential metabolites were subsequently annotated using the Kyoto Encyclopedia of Genes and Genomes database, and pathway enrichment analysis was performed using Fisher’s exact test.

### 2.10. Statistical Analysis

All data were analyzed using one-way ANOVA in SPSS 22.0 (SPSS Inc., Chicago, IL, USA). Multiple comparisons among groups were conducted using the Tukey–Kramer procedure. Differences were regarded as significant when *p* < 0.05. For metabolomic data, principal component analysis and partial least squares discriminant analysis were performed using the R package ropls (version 1.6.2) with seven-cycle interactive validation. Differential metabolites were identified based on VIP > 1 and Student’s *t* test *p* < 0.05. Figures were generated with GraphPad Prism 10.0 (GraphPad Software Inc., San Diego, CA, USA).

## 3. Results

### 3.1. Egg Performance and Egg Quality

Table 2 presents the laying performance and egg quality of hens as affected by dietary 5-ALA supplementation. The egg-laying rate was 2.81% higher in the A250 group than the CON group (*p* < 0.05). No significant differences were observed between the 5-ALA groups and the CON group in average egg weight, shell thickness, shell stiffness, or Haugh unit (*p* > 0.05). Compared with the CON group, albumen height was significantly increased by 13.86% in the A1000 group (*p* < 0.05).

### 3.2. Serum Antioxidant Indicators

Table 3 presents the serum antioxidant indices of laying hens as affected by dietary 5-ALA supplementation. Serum T-AOC was 56.73% higher in the A250 group than the CON group (*p* < 0.05) but did not differ significantly in the A500 or A1000 groups compared to the CON group (*p* > 0.05). Relative to the CON group, GSH-Px activity was increased by an average of 23.81% in the A250, A500, and A1000 groups (*p* < 0.05). Serum MDA concentration and SOD activity were not significantly different among the A250, A500, A1000, and CON groups (*p* > 0.05).

### 3.3. Serum Inflammatory Factor and Immunoglobulin Indicators

Table 4 presents the serum inflammatory factors and immunoglobulin indicators of laying hens as affected by dietary 5-ALA supplementation. Relative to the CON group, serum IgY concentration was increased by an average of 33.04% in the A250, A500, and A1000 groups (*p* < 0.05). Serum IgA concentration was 35.27% higher in the A500 group than in the CON group, whereas no significant differences were observed in the A250 or A1000 groups (*p* > 0.05); serum IgM concentrations were not affected.

Compared with the CON group, serum TNF-α concentration was decreased by 16.18% and 30.48% in the A500 groups but increased in the A1000 group (*p* < 0.05). Relative to the CON group, serum IL-1β and IL-6 concentrations were decreased by 41.03% and 28.48% in the A500 group (*p* < 0.05), whereas no significant differences were observed in the A250 or A1000 groups (*p* > 0.05). Serum IL-10 concentration was increased by 37.69% in the A500 group (*p* < 0.05), while no significant differences were observed in the A250 or A1000 groups (*p* > 0.05).

### 3.4. Liver Antioxidant Indicators of Laying Hens

Table 5 shows the liver antioxidant indicators. MDA content was increased by 43.81% in the A1000 group relative to the CON group (*p* < 0.05). No significant changes were detected in the A250 or A500 groups (*p* > 0.05). T-AOC and SOD activity were higher in the A500 group than the CON group (*p* < 0.05), while no significant changes were detected in the A250 or A1000 groups (*p* > 0.05). In addition, GSH-Px activity was increased by an average of 33.95% in all 5-ALA-supplemented groups compared with the CON group. (*p* < 0.05).

### 3.5. Intestinal Morphology of Laying Hens

**Figure 1:**
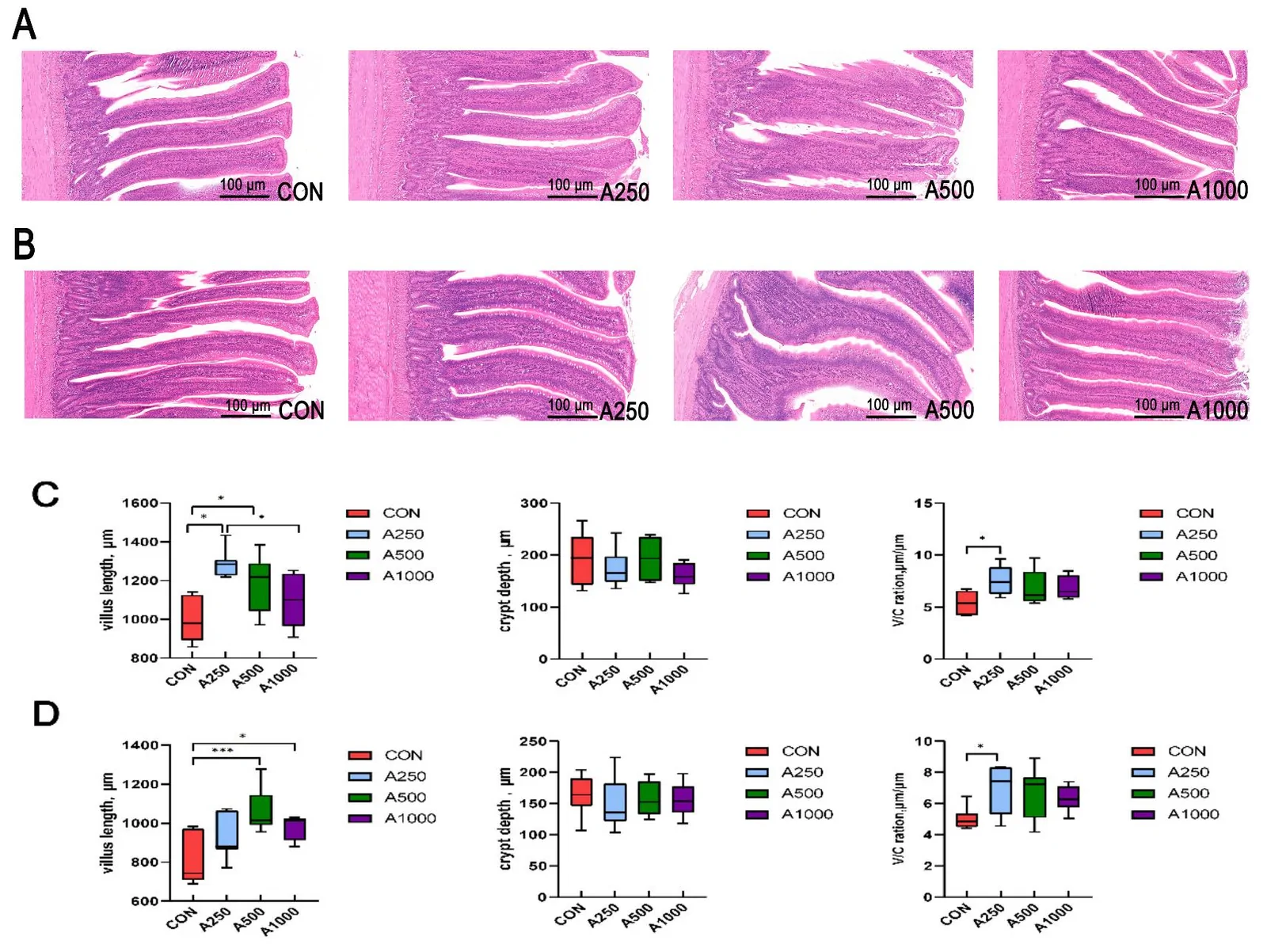
(**A**): Jejunum histomorphometric pictures (H&E staining, 5× magnification). (**B**): ileum histomorphometric pictures (H&E staining, 5× magnification). (**C**): jejunum Statistical analysis of VH, CD and VH/CD. (**D**): ileum Statistical analysis of VH, CD and VH/CD. VH and CD were measured in eight randomly selected villi per slice, and the measurements were averaged to obtain one value for each bird. CON, basal diet; A250, A500, and A1000, basal diet supplemented with 250, 500, and 1000 mg/kg 5-ALA, respectively. * *p* < 0.05, *** *p* < 0.001.

Figure 1 illustrates the jejunum and ileum in laying hens. In the jejunum (Figure 1C), the A250 and A500 groups showed a significant increase in VH and a considerable decrease in CD compared with the CON group. (*p* < 0.05), resulting in a higher VH/CD. No significant differences in jejunal morphological parameters were observed in the A1000 group relative to the CON group (*p* > 0.05).

In the ileum, A250 and A500 supplementation considerably increased VH and VH/CD, while substantially reducing CD relative to the CON group (*p* < 0.05). In contrast, no significant changes in ileal VH, CD, or VH/CD were detected in the A1000 group (*p* > 0.05).

### 3.6. Volatile Fatty Acids of Laying Hens

Table 6 shows the VFA concentrations in laying hens. Relative to the CON group, acetic acid concentration tended to be increased in the A250 and A500 groups (*p* > 0.05), whereas no significant difference was detected in the A1000 group (*p* > 0.05). The levels of propionic acid, isobutyric acid, butyrate, isovaleric acid, and valeric acid were not affected by the 5-ALA group (*p* > 0.05).

### 3.7. Gut Microbiota of Laying Hens

**Figure 2:**
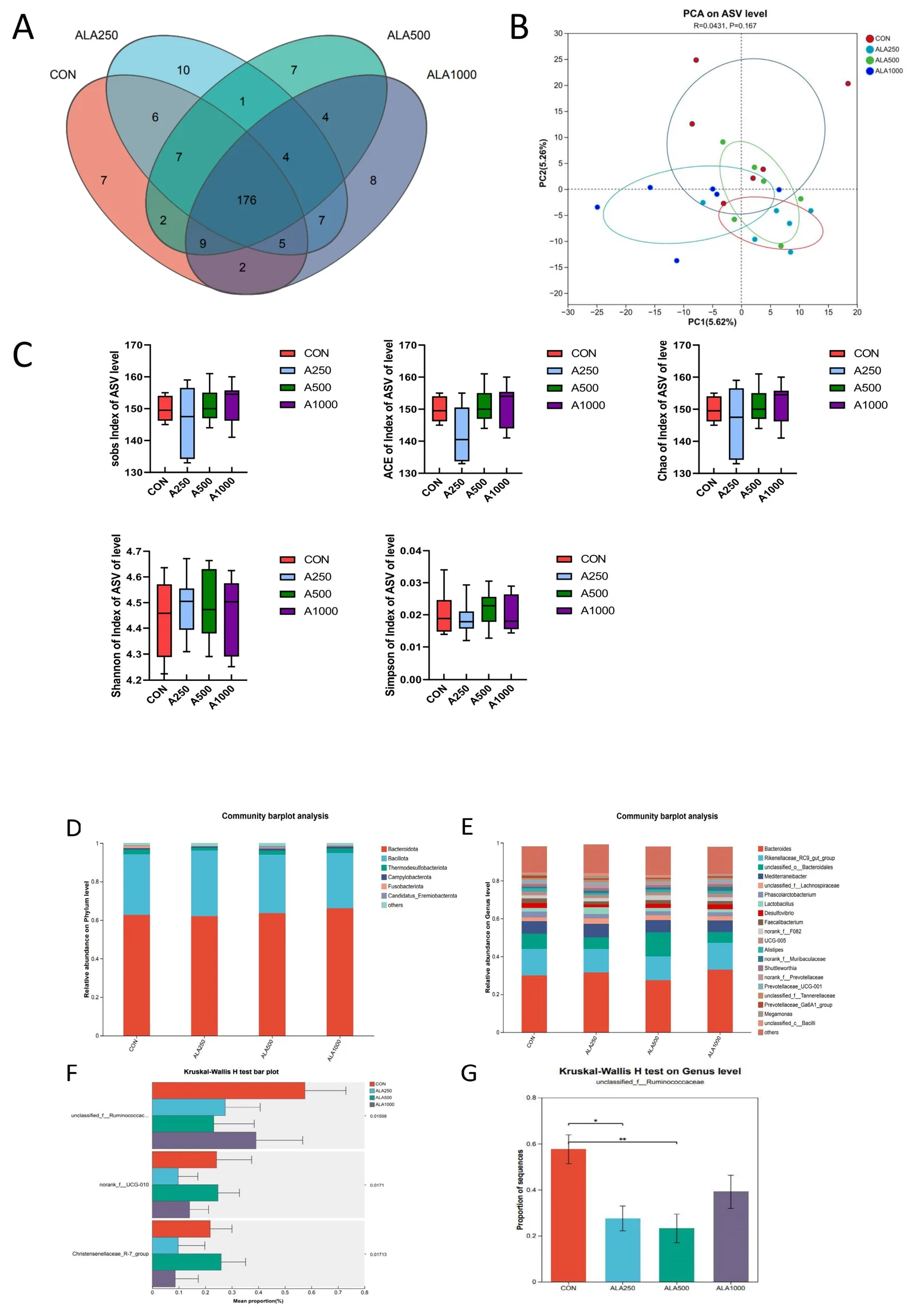
(**A**) Venn diagram of common and unique ASVs in cecal microflora; (**B**) PCA reflecting species beta diversity; (**C**) Alpha diversity indices (Sobs, ACE, Chao, Shannon, Simpson); (**D**) The relative abundance of TOP20 bacteria at the phylum level. (**E**) The relative abundance of TOP20 bacteria at the genus level. (**F**) Kruskal–Wallis H test bar plot of differential microbial taxa at the genus level. (**G**) Genus-level comparison of individual species between groups. CON, basal diet; A250, A500, and A1000, basal diet supplemented with 250, 500, and 1000 mg/kg 5-ALA, respectively. * *p* < 0.05, ** *p* < 0.01.

Figure 2 shows the gut microbial diversity and composition at the amplicon sequence variant (ASV) level. Venn diagram analysis revealed that the majority of ASVs were shared among all groups, while each treatment group also exhibited a small number of unique ASVs compared with the CON group (Figure 2A).

A partial separation between the CON and 5-ALA-supplemented groups was observed by principal component analysis (PCA) (*p* > 0.05) (Figure 2B). The 5-ALA groups showed no significant differences from CON in alpha diversity, as measured by Sobs, ACE, Chao1, Shannon, and Simpson indices. (*p* > 0.05), suggesting that 5-ALA supplementation did not significantly affect microbial richness or diversity (Figure 2C).

*Bacteroidetes* and *Bacillota* were the predominant phyla across all groups, and the community structures were comparable among treatments at both the phylum and genus levels (Figure 2D). However, the abundance of several taxa was significantly affected by 5-ALA supplementation versus the CON group, based on differential abundance analysis (Figure 2F). Specifically, the relative abundance of *Christensenellaceae_R-7_group*, *unclassified_f__ Ruminococcaceae* and *norank_f__UCG-010* was significantly decreased in the 5-ALA group compared with the CON group (*p* < 0.05).

### 3.8. Metabolism of Laying Hens

**Figure 3:**
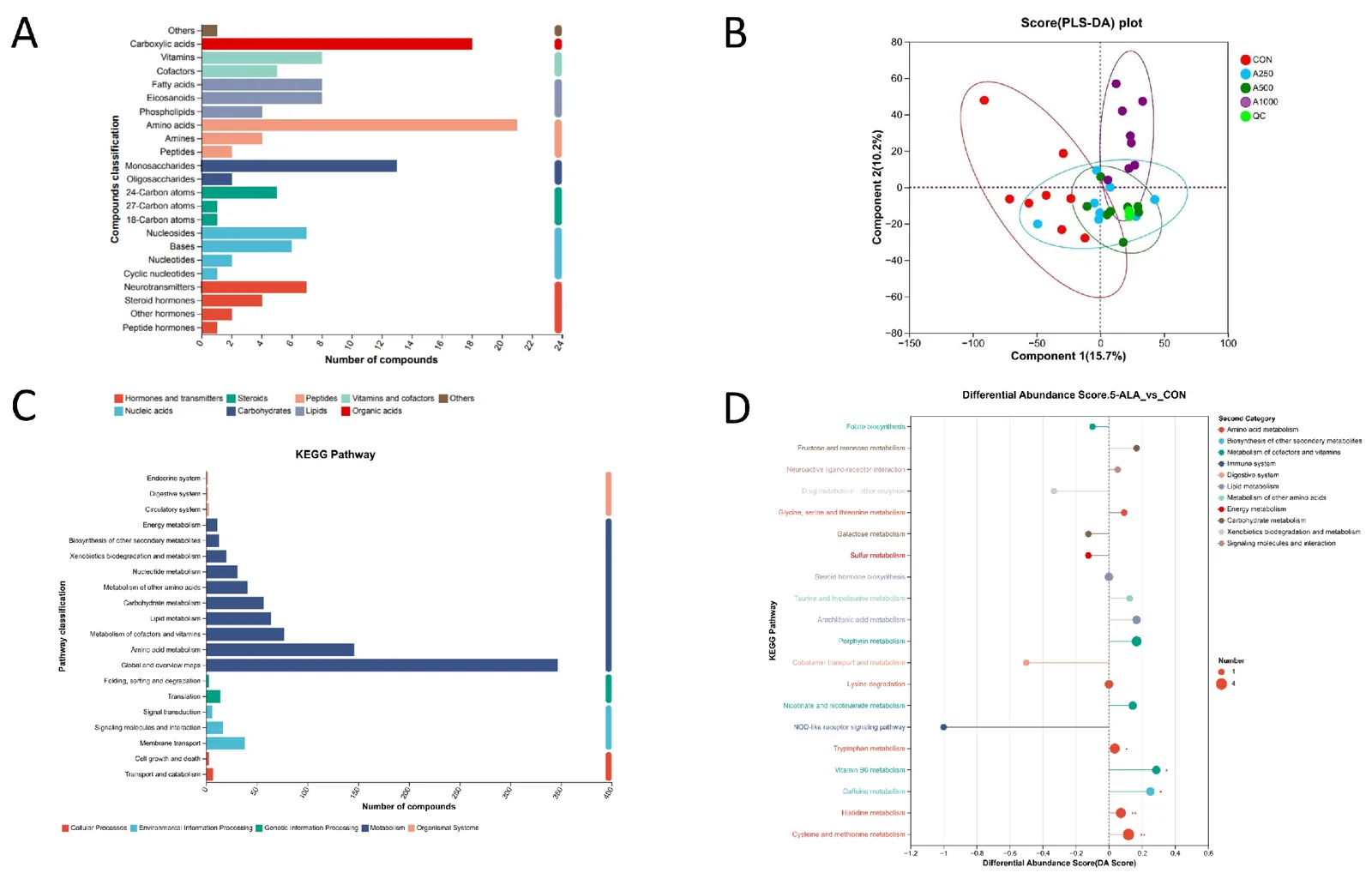
(**A**) Bar chart of KEGG compound classification statistics. The y-axis displays KEGG compound classes, and the x-axis shows compound count per class. Bar color indicates the major category to which each compound is assigned. (**B**) Metabolomic profiles of different groups visualized by Partial least squares discriminant analysis (PLS-DA) score plot. Both axes show relative distances that are inherently quantitative. Each point corresponds to a single sample, colored according to treatment; shorter inter-point distances reflect higher similarity in metabolic composition. (**C**) KEGG pathway classification statistics. The secondary classifications of KEGG pathway subcategories are displayed on the y-axis, with the x-axis showing the number of metabolites annotated per pathway. Bar color corresponds to the primary metabolic pathway category. (**D**) Differential abundance (DA) score plot for KEGG pathways in 5-ALA-treated vs. control laying hens. The x- and y-axes represent DA scores and pathway names, respectively. A DA score reflects the overall regulatory trend of all metabolites mapped to that pathway: 1 indicates an overall upregulation trend and -1 indicates an overall downregulation trend. The length of each line segment corresponds to the absolute DA-score value, and the dot size indicates the number of annotated differential metabolites (larger dots indicating a greater number of differential metabolites). CON, basal diet; A250, A500, and A1000, basal diet supplemented with 250, 500, and 1000 mg/kg 5-ALA, respectively. * *p* < 0.05, ** *p* < 0.01.

Figure 3 illustrates the metabolite profiles of laying hens as affected by 5-ALA supplementation. Across all samples, nine main chemical groups were detected, with amino acids, organic acids, and lipids dominating the profile (Figure 3A). PLS-DA revealed a distinct clustering of the 5-ALA and CON groups, reflecting a shift in the overall metabolic profile of laying hens induced by the dietary supplement (Figure 3B). The analysis identified 20 metabolite-enriched pathways, including amino acid metabolism (146 metabolites), metabolism of cofactors and vitamins (77 metabolites), lipid metabolism (64 metabolites), carbohydrate metabolism (57 metabolites), metabolism of other amino acids (41 metabolites), membrane transport (38 metabolites), nucleotide metabolism (31 metabolites), and xenobiotics biodegradation and metabolism (20 metabolites). Furthermore, metabolites were enriched in signaling molecules and interaction (17 metabolites), translation (14 metabolites), biosynthesis of other secondary metabolites (13 metabolites), and energy metabolism (11 metabolites). Lower numbers of metabolites were associated with transport and catabolism (7 metabolites), signal transduction (6 metabolites), the circulatory system (3 metabolites), folding, sorting and degradation (3 metabolites), cell growth and death (3 metabolites), the endocrine system (2 metabolites), and the digestive system (2 metabolites). The differential abundance score of KEGG pathways is presented in Figure 3D. The five most enhanced pathways, ranked by fold enrichment, were vitamin B6 metabolism, caffeine metabolism, cysteine and methionine metabolism, histidine metabolism, and tryptophan metabolism.

**Figure 4:**
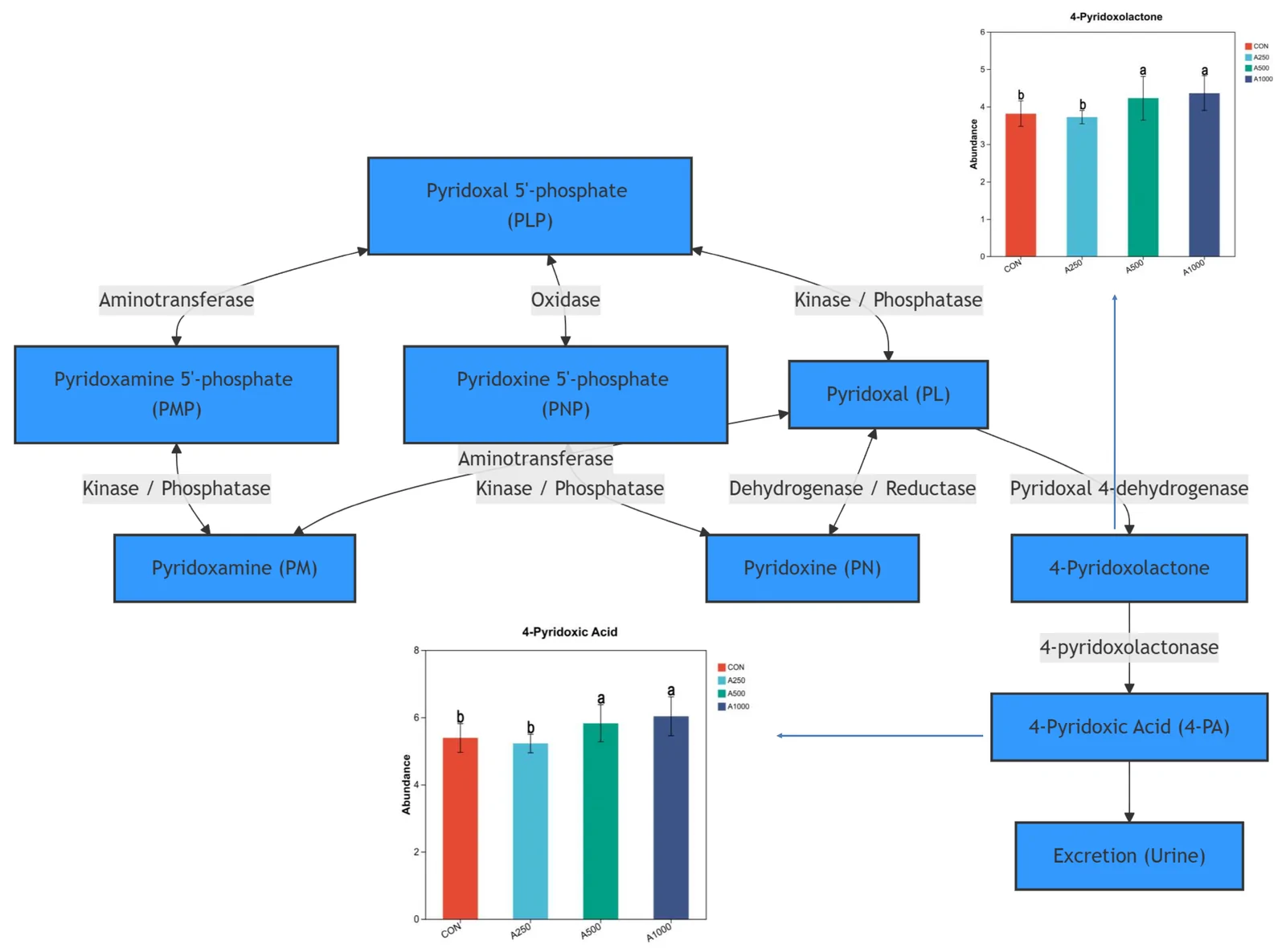
Figure of vitamin B6 metabolism and its intermediate metabolites. Bars represent differentially abundant metabolites in this pathway across treatment groups. The x-axis indicates group, and the y-axis indicates relative abundance. Groups with different lowercase letters (a, b) indicate significant differences at *p* < 0.05. Black arrows indicate the direction of metabolic conversions, while blue arrows point to the bar graphs showing inter-group differences for the corresponding metabolites. CON, basal diet; A250, A500, and A1000, basal diet supplemented with 250, 500, and 1000 mg/kg 5-ALA, respectively.

The vitamin B6 metabolism pathway (map00750) showed the highest differential abundance score among the identified KEGG pathways. Vitamin B6 is a key micronutrient that supports multiple aspects of cellular metabolic activities. It was recognized as a critical enzyme cofactor involved in reactions across various metabolic pathways as early as 1934 [24]. The A500 and A1000 groups exhibited significantly higher levels of 4-pyridoxic acid and 4-pyridoxolactone in this pathway than the control group (*p* < 0.05) (Figure 4).

## 4. Discussion

Poultry production is a major global food sector. In poultry production, laying hens are frequently exposed to various stressors, including immunological challenges, pathogen infections, mycotoxin-contaminated feed, and environmental stress [25]. Non-protein amino acids have garnered considerable attention in recent years. Although they are not incorporated into proteins, they possess diverse biological functions, including metabolic regulation, immune modulation, antioxidant defense, and the promotion of animal growth [26]. 5-ALA has recently drawn attention as an additive for its ability to enhance growth performance and immune function.

Regarding laying performance, Nanto-Hara et al. [27] demonstrated that 100 mg/kg 5-ALA effectively alleviated the decline in egg quality caused by long-term high-temperature exposure. However, Kim JH et al. [28] reported that dietary 5-ALA supplementation (0.25%) had no significant effects on egg weight, eggshell thickness, eggshell strength, or Haugh unit. In the present study, 5-ALA improved laying rate and albumen height, but did not significantly alter egg weight, shell thickness, shell strength, or Haugh unit. These findings are in agreement with previous studies.

Oxidative stress occurs when the production of reactive species overwhelms antioxidant defenses [29]. Key antioxidant enzymes such as SOD and GSH-Px, which are vital for scavenging free radicals, are routinely measured to evaluate antioxidant capacity [30,31]. Heme oxygenase-1 (HO-1) expression is reportedly upregulated by exogenous 5-ALA as a consequence of increased intracellular heme, contributing to the alleviation of oxidative stress [32]. According to Chen et al., GPX1 mRNA and SOD expression were upregulated in all groups receiving 60 mg/kg dietary 5-ALA [33]. Furthermore, Yucong Ye et al. [34] found that dietary 5-ALA upregulated the GPX, GST, CAT, and SOD genes, thereby enhancing the organism’s antioxidant capacity. In this study, T-AOC, SOD, and GSH-Px activities were elevated in laying hens supplemented with 5-ALA, particularly at 500 mg/kg. However, the beneficial effects were attenuated at the higher supplementation level of 1000 mg/kg, which may indicate a dose-dependent response.

Serum IgA, IgY, and IgM are critical indicators of humoral immunity and immune function, given their central roles in infection resistance and host protection [35,36]. Although chickens do not produce mammalian IgG, IgY is regarded as its avian functional equivalent and evolutionary counterpart, with comparable roles in systemic humoral immunity [37]. Yan et al. [38] reported that dietary 5-ALA increased serum IgG concentrations in weaned piglets. Another study by Wang et al. [17] reported that dietary 5-ALA increased B lymphocyte counts in the blood cells of piglets, whereas B lymphocytes secrete immunoglobulins upon antigen stimulation of the immune system. Interestingly, dietary 5-ALA supplementation significantly affected serum IgY and IgA concentrations, whereas IgM levels remained unchanged across the treatment groups. A previous study also reported no significant differences in IgM concentrations among different chicken groups, whereas IgY concentrations differed significantly among these groups [39]. In chickens, IgM is the major immunoglobulin involved in the primary humoral immune response and is produced early following antigen exposure, whereas IgY is predominantly associated with secondary humoral immunity [40]. We speculate that, as an early antibody involved in the primary humoral immune response, IgM may be maintained at a relatively stable basal level in the circulation. These findings suggest that appropriate ALA supplementation enhances immune function in poultry. In this study, dietary 5-ALA significantly raised serum IgY and IgA, with a rising trend in IgM, consistent with prior reports.

Immunometabolic processes in laying hens are critically regulated by cytokines, including TNF-α, IL-6, IL-1β, and IL-10, and their dynamic balance critically influences susceptibility to various diseases as well as overall productive performance [41,42,43]. Sugiyama et al. [44] demonstrated that ALA supplementation reduced TNF-α expression in lipopolysaccharide-stimulated murine macrophages. In agreement with previous findings, both 250 mg/kg and 500 mg/kg of 5-ALA supplementation significantly reduced TNF-α, IL-1β, and IL-6 concentrations and elevated IL-10 levels, with the 500 mg/kg dose exhibiting the most pronounced efficacy. However, the effects were less pronounced at 1000 mg/kg for several parameters.

The intestine functions as the main organ for nutrient uptake while also acting as a critical barrier against pathogenic invasion [45]. Therefore, the integrity of intestinal morphology and function is critical for maintaining poultry health and normal productive performance. VH, CD, and the VH/CD are widely used indicators of intestinal structural integrity [46]. In this study, villus height and the VH/CD ratio in the jejunum and ileum were elevated by optimal dietary 5-ALA, an effect potentially linked to gut microbiota modulation. Consistent with our findings, Pedrosa-Gerasimo et al. [47] reported that dietary 5-ALA reduced intestinal pathogen colonization and increased the relative abundance of beneficial bacteria. In addition, Cheng et al. [48] confirmed that ALA alleviated α-cypermethrin–induced intestinal injury by regulating liver–gut axis-related enzyme activity, enhancing intestinal antioxidant capacity, and restoring microbial balance. Collectively, these findings suggest that appropriate dietary 5-ALA enhances gut morphology and barrier integrity in layers.

The gut microbiota plays essential roles in nutrient metabolism, immune regulation, and intestinal homeostasis [45,49]. In the present study, 5-ALA supplementation did not significantly affect alpha diversity or the overall structure of the cecal microbial community. However, several specific bacterial taxa showed differential abundances among treatments. These findings suggest that dietary 5-ALA may be associated with selective changes in certain bacterial populations rather than broad restructuring of the gut microbiota. Consistent with previous studies, *Bacteroidota* and *Firmicutes* were the predominant phyla in the cecum of laying hens, reflecting their important roles in carbohydrate degradation, energy metabolism, and gastrointestinal homeostasis [50,51,52]. Notably, 5-ALA supplementation reduced the relative abundances of *unclassified_f__Ruminococcaceae* and *Christensenellaceae_R-7_group*. Members of *Ruminococcus*, particularly *Ruminococcus gnavus*, have been reported to degrade intestinal mucins and promote the production of pro-inflammatory cytokines, including IL-1β, IL-6, and TNF-α [53]. Similarly, Christensenellaceae_R-7_group has been reported to be enriched in individuals with intestinal disorders compared with healthy controls [54], whereas dietary vitamin E supplementation reduced its abundance and was associated with improved intestinal health [19]. These findings suggest that, although 5-ALA did not markedly alter overall microbial diversity or VFA production, it may exert a more selective regulatory effect on specific bacterial populations. It is therefore plausible to hypothesize that the reduction of potentially unfavorable taxa, particularly *Ruminococcus*-related bacteria and Christensenellaceae_R-7_group, may contribute to the maintenance of intestinal microbial homeostasis and barrier integrity in laying hens. However, this interpretation remains speculative, and further studies directly evaluating intestinal barrier function and microbial functional activity are needed to determine whether these microbiota changes are mechanistically involved in the intestinal effects of 5-ALA.

Pathway analysis identified vitamin B6 metabolism as the highest differential abundance score, followed by caffeine metabolism, histidine metabolism, and tryptophan metabolism. Vitamin B6 acts as a coenzyme in over 150 metabolic reactions, notably in amino acid and lipid metabolism, and contributes to immune and inflammatory regulation, as shown in animal and human studies [55,56]. In LPS-stimulated immune cells, high concentrations of vitamin B6 attenuated inflammatory responses by reducing IL-1β, IL-6, and TNF-α production, suggesting that enhanced vitamin B6 metabolic activity may help alleviate inflammatory status [57]. Vitamin B6 has recently been shown to downregulate pro-inflammatory cytokine production via suppression of the NF-κB and MAPK pathways, supporting its role in immune regulation [58].

Cysteine and methionine metabolism contributes to glutathione biosynthesis and the production of other sulfur-containing metabolites, thereby playing an important role in maintaining intracellular redox homeostasis [59]. The enrichment of this pathway suggests a potential enhancement of antioxidant defense capacity, which is consistent with previous findings showing that activation of sulfur amino acid metabolism contributes to improved antioxidant and immune functions. In addition, histidine and tryptophan metabolism were substantially enriched. Histidine metabolism is linked to histamine production and immune modulation, whereas tryptophan metabolism regulates intestinal homeostasis and immune responses through its bioactive metabolites [60,61]. Collectively, the coordinated upregulation of these vitamin-dependent and amino acid-related metabolic pathways suggests that dietary 5-ALA may induce adaptive metabolic remodeling. These metabolic changes may contribute to enhanced mitochondrial function, antioxidant capacity, and immune regulation in laying hens.

In eukaryotic cells, 5-ALA is generated from glycine and succinyl-CoA by mitochondrial 5-aminolevulinate synthase (ALAS), and it represents the initial key intermediate in heme biosynthesis [9]. ALAS is an enzyme strictly dependent on pyridoxal 5′-phosphate (PLP) as a cofactor, and its catalytic activity directly depends on the availability of PLP [62]. Studies by Stojanovski et al. have revealed that the catalytic reaction of murine ALAS is reversible, and specific amino acid residues (Asn-150) can regulate the rate balance between the forward and reverse reactions [63]. Based on these findings, we hypothesize that high dietary 5-ALA may be associated with altered PLP utilization or ALAS-related metabolic processes, which could contribute to the changes in vitamin B6 metabolite profiles observed in the present study. A review by Hendawy et al. [64] pointed out that 5-ALA has good antioxidant properties, regulates heme and cytochrome synthesis, and improves body iron content. The exertion of these functions depends to varying degrees on metabolic pathways involving vitamin B6. For example, enhanced heme synthesis may increase the demand for iron, and iron metabolism is related to the activity of the vitamin B6-dependent enzyme aminolevulinic acid dehydratase (ALAD) [65,66]. In the present study, the levels of 4-pyridoxolactone and 4-pyridoxic acid (4-PA), both of which are associated with vitamin B6 metabolism, were significantly increased in the A500 and A1000 groups compared with the CON group. These changes may reflect an alteration in vitamin B6 metabolic status following 5-ALA supplementation. However, the present findings do not establish that high-dose 5-ALA accelerates vitamin B6 metabolism, nor do they demonstrate a direct effect on PLP utilization or ALAS activity. Further studies measuring PLP status, ALAS expression or activity, haem synthesis, and related metabolic intermediates are therefore required to clarify the underlying mechanism.

## 5. Conclusions

Overall, under the present experimental conditions, supplementation with 250 and 500 mg/kg 5-ALA was associated with improvements in several immune-related and antioxidant parameters in laying hens. These changes were accompanied by metabolic characteristics, including changes in vitamin B6-related metabolism.

## Figures and Tables

**Table 1:** Composition and nutrient levels of the control diet (% dry matter).

Items	Content
Ingredients	%
Corn	61.20
Soybean meal	24.80
Soybean oil	1.50
Limestone	8.00
Sodium chloride	0.20
Dicalcium phosphate	11.80
DL-Methionine	10.30
Premix ^1^	2.20
Total	100
Nutrient levels ^2^	/
Metabolizable energy (ME), MJ/kg	10.54
Calcium Crude protein	16.22
Crude protein Lysine	0.85
Available phosphorus Methionine	0.48
Total phosphorus Methionine + cysteine	0.69
Lysine Calcium	4.20
Methionine Total phosphorus	0.72
Methionine + cysteine Available phosphorus	0.45

^1^ The experimental diets were designed to contain, on a per-kilogram basis: 10,000 IU vitamin A; 4000 IU vitamin D_3_; 20 mg vitamin E; 2.5 mg vitamin K_3_; 2.2 mg thiamin; 8 mg riboflavin; 4.5 mg pyridoxine; 0.02 mg cyanocobalamin; 45 mg niacin; 12 mg calcium D-pantothenate; 0.6 mg folic acid; 0.13 mg biotin; and 550 mg choline chloride. Furthermore, trace minerals included: copper (8 mg), manganese (100 mg), iron (70 mg), zinc (80 mg), iodine (0.6 mg), and selenium (0.4 mg). ^2^ ME was estimated by calculation, whereas the other values were acquired through measurement.

**Table 2:** Effects of 5-ALA on egg performance and egg quality of laying hens.

Items	5-ALA, mg/kg				SEM	*p*-Value
	CON	A250	A500	A1000		
Egg laying rate (%)	77.62 ^b^	79.80 ^a^	79.26 ^ab^	78.42 ^ab^	1.053	0.015
Average egg weight (g)	49.29	49.57	49.77	49.93	0.29	0.082
Shell thickness (mm)	0.453	0.455	0.457	0.452	0.001	0.968
Shell stiffness (Kg/cm^2^)	4.097	4.143	4.144	4.202	0.002	0.842
Albumen height (mm)	4.06 ^b^	4.00 ^b^	4.35 ^ab^	4.62 ^a^	0.09	0.019
Haugh Unit (HU)	65.86	64.00	64.39	66.71	0.77	0.67

^a,b^ Means within a row lacking a common superscript differ (*p* < 0.05). CON, basal diet; A250, A500, and A1000, basal diet supplemented with 250, 500, and 1000 mg/kg 5-ALA, respectively.

**Table 3:** Effects of 5-ALA on serum antioxidant indicators of laying hens.

Items	5-ALA, mg/kg				SEM	*p*-Value
	CON	A250	A500	A1000		
MDA (mol/L)	1.77	1.68	1.63	1.75	0.05	0.430
T-AOC (U/mL)	24.57 ^b^	38.51 ^a^	31.47 ^ab^	22.24 ^b^	2.26	0.039
GSH-Px (μmol/L)	669.20 ^c^	786.84 ^b^	848.22 ^a^	856.26 ^a^	13.63	<0.001
SOD (U/mL)	168.96	220.37	235.17	195.68	13.79	0.063

^a,b,c^ Means within a row lacking a common superscript differ (*p* < 0.05).

**Table 4:** Effects of 5-ALA on serum inflammatory factor and immunoglobulin indicators of laying hens.

Items	5-ALA, mg/kg				SEM	*p*-Value
	CON	A250	A500	A1000		
IgY (g/L)	254.98 ^b^	319.70 ^a^	363.66 ^a^	334.36 ^a^	14.25	0.002
IgA (g/L)	108.73 ^b^	116.80 ^b^	147.08 ^a^	131.41 ^ab^	5.96	0.007
IgM (g/L)	87.88	85.86	99.89	95.00	13.63	0.532
TNF-α (ng/mL)	36.08 ^ab^	30.24 ^bc^	25.08 ^c^	44.00 ^a^	1.88	<0.001
IL-1β (ng/mL)	27.96 ^a^	25.22 ^ab^	16.48 ^b^	32.44 ^a^	1.82	0.008
IL-6 (ng/mL)	342.27 ^a^	306.36 ^ab^	244.77 ^b^	331.43 ^a^	12.56	0.020
IL-10 (ng/mL)	11.30 ^b^	12.17 ^ab^	15.56 ^a^	13.49 ^ab^	0.68	0.013

^a,b,c^ Means within a row lacking a common superscript differ (*p* < 0.05). CON, basal diet; A250, A500, and A1000, basal diet supplemented with 250, 500, and 1000 mg/kg 5-ALA, respectively.

**Table 5:** Effects of 5-ALA on liver antioxidant indicators of laying hens.

Items	5-ALA, mg/kg				SEM	*p*-Value
	CON	A250	A500	A1000		
MDA (mol/L)	1.94 ^b^	2.11 ^ab^	1.87 ^b^	2.79 ^a^	0.13	0.043
T-AOC (U/mL)	11.92 ^b^	14.22 ^ab^	16.50 ^a^	14.48 ^ab^	0.71	0.046
GSH-Px(μmol/L)	106.18 ^b^	146.32 ^ab^	167.89 ^a^	136.08 ^ab^	8.18	<0.001
SOD (U/mL)	177.51 ^c^	257.31 ^b^	326.85 ^a^	218.25 ^ab^	14.04	0.022

^a,b,c^ Means within a row lacking a common superscript differ (*p* < 0.05). CON, basal diet; A250, A500, and A1000, basal diet supplemented with 250, 500, and 1000 mg/kg 5-ALA, respectively.

**Table 6:** Effects of 5-ALA on volatile fatty acids of laying hens.

Items	5-ALA, mg/kg				SEM	*p*-Value
	CON	A250	A500	A1000		
Acetic Acid (mg/g)	0.8592	1.0088	1.0923	0.7791	0.0528	0.052
Propionic Acid (mg/g)	0.3863	0.4231	0.4069	0.3025	0.0268	0.161
Isobutyric Acid (mg/g)	0.0536	0.0522	0.0542	0.0533	0.0035	0.507
Butyrate (mg/g)	0.1660	0.1585	0.1819	0.1316	0.0129	0.231
Isovaleric Acid (mg/g)	0.0383	0.0504	0.0582	0.0458	0.0051	0.740
Valeric Acid (mg/g)	0.0414	0.0575	0.0435	0.0409	0.0050	0.513

CON, basal diet; A250, A500, and A1000, basal diet supplemented with 250, 500, and 1000 mg/kg 5-ALA, respectively.

## Data Availability

The sequencing data generated in this study have been deposited in the Sequence Read Archive (SRA) of the National Center for Biotechnology Information (NCBI) and are accessible under accession number PRJNA1457058.

## References

[1] Henchion M., Moloney A.P., Hyland J., Zimmermann J., McCarthy S. (2021). Review: Trends for meat, milk and egg consumption for the next decades and the role played by livestock systems in the global production of proteins. Animal.

[2] Ajijola S., Odetola S., Idowu A.B., Irhivben B., Awoyemi T.T. (2024). Production Challenges of Commercial Laying Hen and Farmers’ Mitigation Strategies. Slovak J. Anim. Sci..

[3] Chen L., Li X., Wu Y., Wang J., Pi J. (2024). Differential analysis of ovarian tissue between high and low-yielded laying hens in the late laying stage and the effect of LECT2 gene on follicular granulosa cells proliferation. Mol. Biol. Rep..

[4] Zhou X., Song Y., Chen J., Chen X., Guan L., Wang Y., Xiao M., Liu W., An L. (2025). Effects of dietary canthaxanthin on egg production, serum parameters, and intestinal health in indigenous chickens under heat stress. Front. Vet. Sci..

[5] Zhao H., Li Z., Sun Y., Yan M., Wang Y., Li Y., Zhang Y., Zhu M. (2024). Supplementation of Chlorogenic Acid Alleviates the Effects of H_2_O_2_-Induced Oxidative Stress on Laying Performance, Egg Quality, Antioxidant Capacity, Hepatic Inflammation, Mitochondrial Dysfunction, and Lipid Accumulation in Laying Hens. Antioxidants.

[6] Chang X., Wang B., Zhang H., Qiu K., Wu S. (2024). The change of albumen quality during the laying cycle and its potential physiological and molecular basis of laying hens. Poult. Sci..

[7] Luo Z., Gong Y., Li Q., Zhang M., Zhang J., Zhang H., Zeng Q., Zhu Y., Guo Y., Li D. (2025). Regulation of intestinal health by Lactobacillus rhamnosus GG during fasting-induced molting in laying hens. Poult. Sci..

[8] Jiang P., Lian M., Liu G., Gu P. (2026). Recent advances in microbial production of 5-aminolevulinic acid by metabolic engineering. 3 Biotech.

[9] Yi Y.C., Shih I.T., Yu T.H., Lee Y.J., Ng I.S. (2021). Challenges and opportunities of bioprocessing 5-aminolevulinic acid using genetic and metabolic engineering: A critical review. Bioresour. Bioprocess..

[10] Fujino M., Nishio Y., Ito H., Tanaka T., Li X.K. (2016). 5-Aminolevulinic acid regulates the inflammatory response and alloimmune reaction. Int. Immunopharmacol..

[11] Zhu Y., Hon T., Ye W., Zhang L. (2002). Heme deficiency interferes with the Ras-mitogen-activated protein kinase signaling pathway and expression of a subset of neuronal genes. Cell Growth Differ..

[12] Jiang M., Hong K., Mao Y., Ma H., Chen T., Wang Z. (2022). Natural 5-Aminolevulinic Acid: Sources, Biosynthesis, Detection and Applications. Front. Bioeng. Biotechnol..

[13] Li J., Xie Y., Li M., Zhang S., Cheng Q., Yang W. (2022). Effects of Feeding 5-Aminolevulinic Acid on Iron Status in Weaned Rats from the Female Rats during Gestation and Lactation. Animals.

[14] Hossain M.M., Park J.W., Kim I.H. (2016). δ-Aminolevulinic acid, and lactulose supplements in weaned piglets diet: Effects on performance, fecal microbiota, and in-vitro noxious gas emissions. Livest. Sci..

[15] Wang J.P., Yan L., Lee J.H., Zhou T.X., Kim I.H. (2011). Effects of dietary delta-aminolevulinic acid and vitamin C on growth performance, immune organ weight and ferrum status in broiler chicks. Livest. Sci..

[16] Sato K., Matsushita K., Takahashi K., Aoki M., Fuziwara J., Miyanari S., Kamada T. (2012). Dietary supplementation with 5-aminolevulinic acid modulates growth performance and inflammatory responses in broiler chickens. Poult. Sci..

[17] Wang J.P., Lee J.H., Jang H.D., Yan L., Cho J.H., Kim I.H. (2011). Effects of δ-aminolevulinic acid and vitamin C supplementation on iron status, production performance, blood characteristics and egg quality of laying hens. J. Anim. Physiol. Anim. Nutr..

[18] Dale N. (1994). National Research Council Nutrient Requirements of Poultry—Ninth Revised Edition (1994). J. Appl. Poult. Res..

[19] Wang Y., Xue Y., Yan C., Yu X., Zhang L., Wang Y., Lan Y., Zhang X. (2024). Ovary metabolome and cecal microbiota changes in aged laying hens supplemented with vitamin E. Poult. Sci..

[20] Chen L., Peng W., Wang T., Ma S., Wang X., Dai B., Zhang R., Yang C., Wu Y. (2025). Effects of dietary lysozyme supplementation on growth performance, intestinal morphology, immune function, antioxidant capacity, and gut microbiota in broilers. Poult. Sci..

[21] Wang T., Xu H., Zhang S., Hu Y., Wu Y., Zhang R., Xiao X., Yang C. (2025). Effect of lysozyme supplementation on growth performance, immune status, antioxidant function and intestinal microbiota in broilers. Poult. Sci..

[22] Cuccato M., Rubiola S., Giannuzzi D., Grego E., Pregel P., Divari S., Cannizzo F.T. (2021). 16S rRNA Sequencing Analysis of the Gut Microbiota in Broiler Chickens Prophylactically Administered with Antimicrobial Agents. Antibiotics.

[23] Xu H., Wang X., Peng W., Hu Y., Xu Y., Xiao X., Dai B., Zhang R., Zhong Y., Yang C. (2025). Dietary Pyrroloquinoline Quinone Addition Alleviated Weaning Stress via Modulation of Gut Microbiota and Metabolic Profiles in Weaned Piglets. Animals.

[24] Rosenberg I.H. (2012). A history of the isolation and identification of vitamin B_6_. Ann. Nutr. Metab..

[25] Ncho C.M., Berdos J.I., Gupta V., Rahman A., Mekonnen K.T., Bakhsh A. (2025). Abiotic stressors in poultry production: A comprehensive review. J. Anim. Physiol. Anim. Nutr..

[26] Zou Y., Jiang X., Li N., Zhong S., Zhang S., Ji Y., Yu H., Zeng X., Deng A., Qiao S. (2026). Recent advances in nonprotein amino acids: Insights from function to biosynthesis. J. Anim. Sci. Biotechnol..

[27] Nanto-Hara F., Ohtsu H. (2025). Dietary 5-Aminolevulinic Acid Alleviates Heat Stress-Induced Renal Injury in Laying Hens by Improving Mitochondrial Quality and Enhancing Antioxidant Activity. Antioxidants.

[28] Kim J.H., Choi W.J., Kwon C.H., Kil D.Y. (2022). Research Note: Improvement of eggshell strength and intensity of brown eggshell color by dietary magnesium and δ-aminolevulinic acid supplementation in laying hens. Poult. Sci..

[29] Birben E., Sahiner U.M., Sackesen C., Erzurum S., Kalayci O. (2012). Oxidative stress and antioxidant defense. World Allergy Organ. J..

[30] Jena A.B., Samal R.R., Bhol N.K., Duttaroy A.K. (2023). Cellular Red-Ox system in health and disease: The latest update. Biomed. Pharmacother..

[31] Shahidi F., Samarasinghe A. (2025). How to assess antioxidant activity? Advances, limitations, and applications of in vitro, in vivo, and ex vivo approaches. Food Prod. Process. Nutr..

[32] Zhao M., Zhu P., Fujino M., Nishio Y., Chen J., Ito H., Takahashi K., Nakajima M., Tanaka T., Zhao L. (2016). 5-Aminolevulinic acid with sodium ferrous citrate induces autophagy and protects cardiomyocytes from hypoxia-induced cellular injury through MAPK-Nrf-2-HO-1 signaling cascade. Biochem. Biophys. Res. Commun..

[33] Chen J., Wang H., Wu Z., Gu H., Li C., Wang S., Liu G. (2022). Effects of 5-aminolevulinic acid on the inflammatory responses and antioxidative capacity in broiler chickens challenged with lipopolysaccharide. Animal.

[34] Ye Y., Li S., Du X., Zhang L., Bao N., Li Y., Zhao Y. (2024). Effects of dietary 5-aminolevulinic acid on growth performance and nonspecific immunity of Litopenaeus vannamei, as determined by transcriptomic analysis. Fish Shellfish Immunol..

[35] Olaniyan M.F., Muhibi M.A. (2024). Immunoglobulins as Tools for Passive Immunization in Preventive Healthcare. J. Prim. Care Spec..

[36] Sun Y., Liu Z., Ren L., Wei Z., Wang P., Li N., Zhao Y. (2012). Immunoglobulin genes and diversity: What we have learned from domestic animals. J. Anim. Sci. Biotechnol..

[37] Garcia P., Wang Y., Viallet J., Macek Jilkova Z. (2021). The Chicken Embryo Model: A Novel and Relevant Model for Immune-Based Studies. Front. Immunol..

[38] Yan L., Kim I.H. (2011). Evaluation of dietary supplementation of delta-aminolevulinic acid and chitooligosaccharide on growth performance, nutrient digestibility, blood characteristics, and fecal microbial shedding in weaned pigs. Anim. Feed Sci. Technol..

[39] Khazali D., Ehsani A., Masoudi A.A., Torshizi M.A.K., Torshizi R.V., Davoodi P., Valizadeh A., Sheykh-Al-Eslami S.M.A. (2025). Comparison of Immune-Related Responses of Several Slow-Growing Indigenous Chickens with a Fast-Growing Broiler in Iran. Vet. Med. Sci..

[40] Parra D., Takizawa F., Sunyer J.O. (2013). Evolution of B cell immunity. Annu. Rev. Anim. Biosci..

[41] Xu Z.-Y., Yu Y., Liu Y., Ou C.-B., Zhang Y.-H., Liu T.-Y., Wang Q.-X., Ma J.-Y. (2019). Differential expression of pro-inflammatory and anti-inflammatory genes of layer chicken bursa after experimental infection with infectious bursal disease virus. Poult. Sci..

[42] Jing M., Li S., Zhao S., House J.D. (2024). Splenic gene expression of cytokines at multiple time points following lipopolysaccharide challenge in layers. Am. J. Vet. Res..

[43] Liu C., Chu D., Kalantar-Zadeh K., George J., Young H.A., Liu G. (2021). Cytokines: From Clinical Significance to Quantification. Adv. Sci..

[44] Sugiyama Y., Hiraiwa Y., Hagiya Y., Nakajima M., Tanaka T., Ogura S.I. (2018). 5-Aminolevulinic acid regulates the immune response in LPS-stimulated RAW 264.7 macrophages. BMC Immunol..

[45] Aminullah N., Langar H., Mahaq O., Azizi M.N., Zahir A. (2026). Microbial ecology, functional implications, and associated factors influencing poultry intestinal health. Vet. Immunol. Immunopathol..

[46] Belote B.L., Soares I., Sanches A.W.D., de Souza C., Scott-Delaunay R., Lahaye L., Kogut M.H., Santin E. (2023). Applying different morphometric intestinal mucosa methods and the correlation with broilers performance under Eimeria challenge. Poult. Sci..

[47] Pedrosa-Gerasmio I.R., Nozaki R., Kondo H., Hirono I. (2020). Gut bacterial community profile in Pacific white shrimp Litopenaeus vannamei following 5-aminolevulinic acid supplementation. Aquac. Res..

[48] Cheng Y., Zhang J., Gao F., Xu Y., Wang C. (2022). Protective effects of 5-aminolevulinic acid against toxicity induced by alpha-cypermethrin to the liver-gut-microbiota axis in zebrafish. Ecotoxicol. Environ. Saf..

[49] Liu H., Lu H., Wang Y., Yu C., He Z., Dong H. (2024). Unlocking the power of short-chain fatty acids in ameliorating intestinal mucosal immunity: A new porcine nutritional approach. Front. Cell. Infect. Microbiol..

[50] Rychlik I. (2020). Composition and Function of Chicken Gut Microbiota. Animals.

[51] Aruwa C.E., Pillay C., Nyaga M.M., Sabiu S. (2021). Poultry gut health—Microbiome functions, environmental impacts, microbiome engineering and advancements in characterization technologies. J. Anim. Sci. Biotechnol..

[52] Utkina I., Fan Y., Willing B.P., Parkinson J. (2025). Metabolic modeling of microbial communities in the chicken ceca reveals a landscape of competition and co-operation. Microbiome.

[53] Laplanche V., Armiento S., Speciale I., Šuligoj T., Crost E.H., Lamprinaki D., Vaux L., Gotts K., De Castro C., Juge N. (2025). The human gut symbiont *Ruminococcus gnavus* displays strain-specific exopolysaccharides modulating the host immune response. Carbohydr. Polym..

[54] Mancabelli L., Milani C., Lugli G.A., Turroni F., Cocconi D., van Sinderen D., Ventura M. (2017). Identification of universal gut microbial biomarkers of common human intestinal diseases by meta-analysis. FEMS Microbiol. Ecol..

[55] Stach K., Stach W., Augoff K. (2021). Vitamin B6 in Health and Disease. Nutrients.

[56] Ueland P.M., McCann A., Midttun Ø., Ulvik A. (2017). Inflammation, vitamin B6 and related pathways. Mol. Asp. Med..

[57] Mikkelsen K., Dargahi N., Fraser S., Apostolopoulos V. (2023). High-Dose Vitamin B6 (Pyridoxine) Displays Strong Anti-Inflammatory Properties in Lipopolysaccharide-Stimulated Monocytes. Biomedicines.

[58] Du X., Yang Y., Zhan X., Huang Y., Fu Y., Zhang Z., Liu H., Zhang L., Li Y., Wen Q. (2020). Vitamin B6 prevents excessive inflammation by reducing accumulation of sphingosine-1-phosphate in a sphingosine-1-phosphate lyase-dependent manner. J. Cell. Mol. Med..

[59] Bin P., Huang R., Zhou X. (2017). Oxidation Resistance of the Sulfur Amino Acids: Methionine and Cysteine. Biomed. Res. Int..

[60] Moriguchi T., Takai J. (2020). Histamine and histidine decarboxylase: Immunomodulatory functions and regulatory mechanisms. Genes Cells.

[61] Li Y., Liu N., Ge Y., Yang Y., Ren F., Wu Z. (2022). Tryptophan and the innate intestinal immunity: Crosstalk between metabolites, host innate immune cells, and microbiota. Eur. J. Immunol..

[62] Hunter G.A., Ferreira G.C. (2011). Molecular enzymology of 5-aminolevulinate synthase, the gatekeeper of heme biosynthesis. Biochim. Biophys. Acta.

[63] Stojanovski B.M., Ferreira G.C. (2015). Asn-150 of Murine Erythroid 5-Aminolevulinate Synthase Modulates the Catalytic Balance between the Rates of the Reversible Reaction. J. Biol. Chem..

[64] Hendawy A.O., Khattab M.S., Sugimura S., Sato K. (2020). Effects of 5-Aminolevulinic Acid as a Supplement on Animal Performance, Iron Status, and Immune Response in Farm Animals: A Review. Animals.

[65] Testa U., Pelosi E., Castelli G. (2025). Outline of Iron Metabolism, with Emphasis on Erythroid Cells. Mediterr. J. Hematol. Infect. Dis..

[66] Ponka P., Zhang A.-S., Sheftel A., Shirihai O.S. (2008). The Path of Iron from Plasma Transferrin to Hemoglobin in Developing Red Blood Cells. Blood.

